# Quality of reporting of systematic reviews and meta-analyses in emergency medicine based on the PRISMA statement

**DOI:** 10.1186/s12873-019-0233-6

**Published:** 2019-02-11

**Authors:** Femke Nawijn, Wietske H. W. Ham, Roderick M. Houwert, Rolf H. H. Groenwold, Falco Hietbrink, Diederik P. J. Smeeing

**Affiliations:** 1 Department of Surgery, University Medical Center Utrecht, Utrecht, the Netherlands; 2 Department of acute care education, University of Applied Science, Utrecht, the Netherlands; 3 Department of Clinical Epidemiology, Leiden University Medical Center, Leiden, the Netherlands

**Keywords:** Epidemiology, Quality of reporting, Systematic review, Meta-analysis, Emergency medicine

## Abstract

**Background:**

Emergency department utilization has increased tremendously over the past years, which is accompanied by an increased necessity for emergency medicine research to support clinical practice. Important sources of evidence are systematic reviews (SRs) and meta-analyses (MAs), but these can only be informative provided their quality is sufficiently high, which can only be assessed if reporting is adequate. The purpose of this study was to assess the quality of reporting of SRs and MAs in emergency medicine using the PRISMA statement.

**Methods:**

The top five emergency medicine related journals were selected using the 5-year impact factor of the ISI Web of Knowledge of 2015. All SRs and MAs published in these journals between 2015 and 2016 were extracted and assessed independently by two reviewers on compliance with each item of the PRISMA statement.

**Results:**

The included reviews (*n* = 112) reported a mean of 18 ± 4 items of the PRISMA statement adequately. Reviews mentioning PRISMA adherence did not show better reporting than review without mention of adherence (mean 18.6 (SE 0.4) vs. mean 17.8 (SE 0.5); *p* = 0.214). Reviews published in journals recommending or requiring adherence to a reporting guideline showed better quality of reporting than journals without such instructions (mean 19.2 (SE 0.4) vs. mean 17.2 (SE 0.5); *p* = 0.001).

**Conclusion:**

There is room for improvement of the quality of reporting of SRs and MAs within the emergency medicine literature. Therefore, authors should use a reporting guideline such as the PRISMA statement. Active journal implementation, by requiring PRISMA endorsement, enhances quality of reporting.

**Electronic supplementary material:**

The online version of this article (10.1186/s12873-019-0233-6) contains supplementary material, which is available to authorized users.

## Background

Over the past years, a tremendous increase in emergency department utilization has been seen. This is caused by rapid urbanization, aging of the population, and changes in population morbidity [1, 2]. Which results in an urgent need for a solid evidence base to meet up with the increased demand for emergency care, and to support clinical practice and the providence of the most optimal care possible [3]. The evidence base that is provided by systematic reviews (SRs) and meta-analyses (MAs) is often considered to be of the highest level. They facilitate an efficient way for clinicians to keep up-to-date with the current state of evidence and provide a starting point for development of clinical practice guidelines [4–7]. Still, caution must be taken given that SRs and MAs are affected by the flaws in the included studies, as well as the quality of the execution of the review itself [8]. Therefore, adequate reporting of the methodology, the results, and the risk of bias is essential for evaluating the strengths and weaknesses of the evidence provided [5, 9]. Clear, complete, and transparent reported research aids reproducibility and critical appraisal [10]. Therefore, the PRISMA statement was formulated to address the problem of suboptimal reporting. This is a reporting guideline suitable for both SRs and MAs [5, 11]. Previous conducted studies found that journals endorsing the PRISMA statement publish SRs and MAs that are more complete and of higher quality [12–14].

Since emergency medicine is a relative new specialty, including the field of emergency medicine research, the number of emergency medicine related SRs and MAs is still limited [3]. Given the increased necessity and demand for emergency medicine research, it is of the utmost importance that the quality of reporting of the available reviews is high to facilitate the providence of high-level evidence. Evaluating the quality of reporting enables interpretation of the current quality of evidence and thus the current state and clinical relevancy. This insight helps to draft a research agenda for the near future within the highly demanding field of emergency medicine. The quality of reporting of SRs and MAs has been accessed within different medical fields [15–17]. However, until now it has not been evaluated within the field of emergency medicine. This resulted in our objective to assess the quality of reporting of SRs and MAs in emergency medicine using the PRISMA statement.

## Methods

Institutional Review Board approval was not obtained and deemed unnecessary as the study did not involve human participants. A study protocol was not registered or published. Prior to execution of this study, the search strategy and data extraction procedures were defined. No amendments were made during study execution. If applicable, this meta-analytical study was written in compliance with the PRISMA statement.

### Search and study selection

The 5-year impact factor (5-YIF) of 2015, based on the Journal Citation Reports Science Edition of the ISI Web of Knowledge (http://www.webofknowledge.com), was used to identify the top five journals related to emergency medicine at that time. From these journals, all reviews from 2015 and 2016 were identified independently by two reviewers (FN, DS) by searching the content list of all volumes published in 2015 and 2016. After screening titles and abstracts, full-text articles were retrieved to identify SRs and MAs.

Eligibility criteria for inclusion were defined a priori. To be eligible for inclusion, studies had to be a SR or MA. The definition of SRs and MAs was adopted from the Cochrane Collaboration (http://handbook.cochrane.org), which is also referred to by the PRISMA Statement: “A systematic review is a review of a clearly formulated question that uses systematic and explicit methods to identify, select, and critically appraise relevant research, and to collect and analyze data from the studies that are included in the review. Statistical methods (meta-analysis) may or may not be used to analyze and summarize the results of the included studies. Meta-analysis refers to the use of statistical techniques in a systematic review to integrate the results of included studies” [5]. Additionally, the reviews had to fulfill the following criteria: reviews were published in English and the full-text review could be obtained. Protocols, reviews in short, review snapshots, scoping reviews and explanatory, nonsystematic, narrative reviews were excluded. Furthermore, the decision was made to include only clinical SRs and MAs to increase comparability between reviews, as the PRISMA statement mainly focuses on the reporting of reviews evaluating randomized trials [5, 11]. This resulted in exclusion of methodological reviews, policy reviews, ethical review, health economic evaluation and animal studies. Eligibility was assessed independently by both reviewers (FN, DS). Discrepancies between reviewers were discussed until consensus was reached. If no consensus could be reached, a third reviewer (FH) was consulted to reach consensus.

### Data collection and assessment of adherence to the PRISMA statement

For all included reviews the following characteristics were obtained: type of review (SR or MA), year of publication, journal, if the author stated that the review was written in adherence to the PRISMA statement, number of included articles in the review, if only randomized controlled trials (RCTs) were included In the review, if any of the authors was affiliated with a department of epidemiology or statistics, number of authors and country of origin (based on first author). All journals were contacted for inquiry to determine if PRISMA adherence was a requirement in their instructions for authors prior to 2015.

All the included reviews were read and scored by assessing the compliance with the PRISMA statement [5]. The PRISMA statement consists of a 27 item checklist to help authors with transparent reporting of SRs and MAs [5, 11]. Of the 27 items, one item assess the reporting of the title, one item assess the abstract, two items assess introduction, twelve items assess the methods, seven items assess the results, three assess the discussion and one assess reporting of funding. The article by Liberati et al. with the explanation and elaboration formulated by the PRISMA group was used to assess whether an item was reported adequately [11]. The criteria from the PRISMA statement are elaborated in Additional file 1. This checklist was pre-specified after discussion and consensus between two reviewers (FN, DS). We emphasize that reviews did not have to present all items in a specific order or section, as long as the information for an item was reported adequately in the review [11]. It was, however, pre-specified that PRISMA items 5 through 16 had to be mentioned under the subheading “methods”, due to the importance of an elaborate method section. Reporting of the items was categorized as reported adequately, not reported adequately, or not applicable. Item 16 and 23 were not applicable if the review concerned a SR. Furthermore, reporting of item 19, 22, and 23 was assessed as not applicable if earlier mentioned, in the methods section, that this assessment or analysis would not be performed. If elaboration of one of the items was sufficiently done in an appendix or a protocol to which was correctly referred, the item was assessed as adequately reported. All reviews were assessed independently by two reviewers (FN, DS). Differences in opinion were discussed until consensus was reached. If no consensus could be reached, a third reviewer (FH) was consulted to reach consensus.

### Data analysis

The sum of adequately reported items in the PRISMA statement was used to measure the overall compliance with the PRISMA statement. Continuous data were presented as means with standard deviations (SD) if normally distributed, or standard error (SE) in cases of adjusted means, and as median with interquartile range (IQR) if non-normal distributed. Dichotomous data were presented as frequencies with percentages. If the review had certain items scored as not applicable, this was defined as a missing variable in the analysis. Missing data were handled using pairwise deletion. In all analyses, correcting for the type of study (SR or MA) was deemed necessary, since SRs could only score a maximum of 25 points compared to the maximum of 27 points for MAs. A priori was decided to compare the overall compliance of reviews with mention of PRISMA adherence and without mention. Further subgroup analyses were performed assessing the overall compliance between reviews published in journal with and without requiring endorsement of a reporting guideline for SRs and MAs in their reporting guideline, between reviews only including RCTs and reviews not limited to RCTs and between reviews with and without an author who has an affiliation to a department of epidemiology or statistics. Continuous variables (overall compliance) were compared using the ANCOVA test with correction for type of review (SR or MA). A priori was decided to analyze the differences per statement item between reviews mentioning PRISMA use and those without by using the Cochran-Mantel-Haenszel test with type of review (SR or MA) as covariate. If the homogenous association assumption for this test was violated (Breslow-Day with a *p*-value > 0.05), a logistic regression with correction for type of review (SR or MA) was performed. If the overall compliance to the PRISMA statement was statistically significant in the subgroup analyses, then the differences per statement item for that factor was also assessed. For all analyses, a two-sided *p*-value < 0.05 was considered statistically significant. All statistical analyses were performed using SPSS (IBM Corp. Released 2017. IBM SPSS Statistics for Windows, version 25.0. Armonk, NY: IBM Corp.).

## Results

### Journal selection, search and study selection

The top 5 journals related to emergency medicine were ‘*Annals of Emergency Medicine’* (5-YIF = 5.244) ‘*Resuscitation’* (5-YIF = 4.991), ‘*Academic Emergency Medicine’* (5-YIF = 2.816), ‘*Scandinavian Journal of Trauma, Resuscitation and Emergency Medicine’* (5-YIF = 2.629) and ‘*Injury’* (5-YIF = 2.408). *Academic Emergency Medicine, Annals of Emergency Medicine* and *Resuscitation* required authors to use the PRISMA statement prior to 2015. At that time, *Scandinavian journal of trauma, resuscitation and emergency medicine* and *Injury* did not make such requirements in their author instructions for SRs and MAs.

**Fig. 1:**
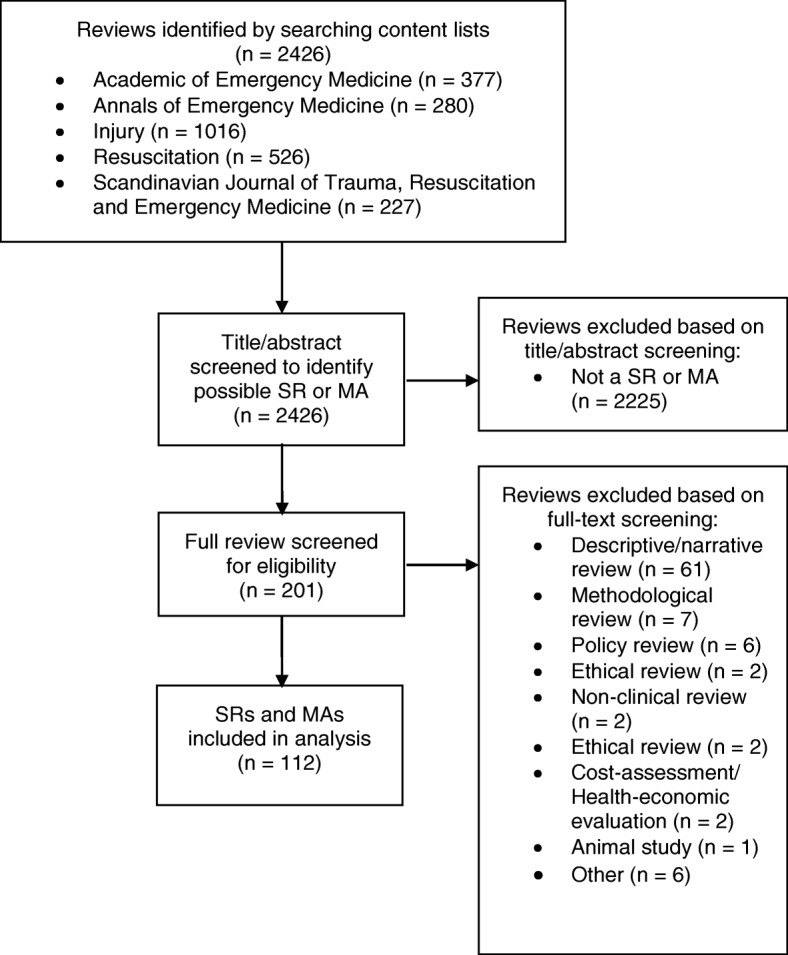
Flowchart of study selection process

A total of 112 reviews published in 2015 and 2016 were included. All other published articles (*n* = 2314) were excluded based on title, abstract and/or full-text (Fig. 1). Of the included reviews, 52 (46%) were published in 2015 and 60 (54%) in 2016. Out of the 112 reviews, 54 reviews were SRs (48%) and 58 were MAs (52%). Sixty-seven reviews mentioned to be written in adherence to the PRISMA statement (60%), the other 45 reviews had no such mention of the PRISMA statement (40%). Fifteen reviews (13%) only included RCTs in their review and 13 reviews (12%) had an author affiliated to an epidemiology or statistic department. An overview of the summary baseline characteristics can be found in Table 1, a complete overview is presented in Additional file 2 and all included reviews are referenced in Additional file 3.

**Table 1:** Summary baseline characteristics of the included reviews

	Total *n* = 112
Type of review, n (%)	
Systematic review	54 (48)
Meta-analysis	58 (52)
Year of publication, n (%)	
2015	52 (46)
2016	60 (54)
Journal, n (%)	
AcEM	21 (19)
AEM	10 (9)
Injury	36 (32)
Resuscitation	32 (28)
SJTREM	13 (12)
Published in journal requiring PRISMA adherence, n (%)	63 (56)
PRISMA use mentioned in review, n (%)	67 (60)
Number of articles included in review, median (IQR)	5 (4–6)
Only RCT’s included in the review, n (%)	15 (13)
Author with affiliation to an epidemiology and/or statistic department, n (%)	13 (12)

*AcEM* Academic Emergency Medicine, *AEM* Academic Emergency Medicine, *IQR* InterQuartile Range, *PRISMA* preferred reporting items for systematic reviews and meta-analyses, *RCT* randomized controlled trial, *SJTREM* Scandinavian Journal of Trauma, Resuscitation and Emergency Medicine

### PRISMA statement adherence

The detailed assessment of every included review, based on all PRISMA items, can be found in Additional file 4. None of the included reviews fulfilled all criteria of the PRISMA Statement. Overall, the included reviews reported a mean of 18 ± 4 items out of 27 items adequately (Table 2). The scoring item all reviews performed lowest on was adequate reporting on the existence of a protocol and/or where it can be found (19%). Furthermore, most reviews failed to adequately report the reasons for exclusion at each stage during the process of study selection (26%). Most reviews did not report the intention and/or results of any assessment of publication bias (26 and 27%, respectively). Reporting on the rationale (100%), the main results (99%) and the summary of evidence (98%) were executed best by all reviews (Table 3).

**Table 2:** The influence of different factors on the overall PRISMA checklist adherence adjusted for type or review

		F -value	*p -* value^a^
Total of all review, unadjusted mean ± SD	18 ± 4	NA	NA
Published in journal requiring PRISMA adherence, adjusted mean (SE)			
Yes	19.2 (0.4)	11.0	0.001
No	17.2 (0.5)		
PRISMA use mentioned in review, adjusted mean (SE)			
Yes	18.6 (0.4)	1.6	0.214
No	17.8 (0.5)		
Type of article included in review, adjusted mean (SE)			
Only RCT’s	18.2 (0.8)	0.5	0.486
Not limited to RCT’s	18.8 (0.3)		
Author with affiliation to a epidemiology and/or statistic department, adjusted mean (SE)			
Yes	19.9 (0.9)	3.7	0.057
No	18.1 (0.3)		

*NA* not applicable, *PRISMA* preferred reporting items for systematic reviews and meta-analyses, *RCT* randomized controlled trial, *SD* standard deviation, *SE* standard error

^a^ANCOVA correcting for type of article (systematic review or meta-analysis)

**Table 3:** Factors influencing the adherence to each PRISMA item adjusted for type of review

	Total adherence *n* = 112 (100%)	PRISMA mention vs. No PRISMA mention in article *p* – value^a^	Journal requiring PRISMA adherence vs. no journal requirement *p* - value^a^
1. Title	93 (83)	0.053	0.607
2. Structured summary	104 (93)	0.887	0.192
3. Rationale	112 (100)	NA	NA
4. Objectives	100 (89)	0.796	0.118
5. Protocol and registration	21 (19)	0.128	0.954
6. Eligibility criteria	104 (93)	0.622^b^	0.542
7. Information sources	96 (86)	0.301	0.571
8. Search	59 (53)	0.270	0.091
9. Study selection	90 (80)	0.205	0.020^2^
10. Data collection process	64 (57)	0.431	0.130
11. Data items	75 (67)	0.701^b^	0.909
12. Risk of bias in individual studies	87 (78)	0.352	0.027^b.2^
13. Summary measures	63 (56)	0.851	0.960
14. Synthesis of results	79 (71)	0.908	0.116
15. Risk of bias across studies	29 (26)	0.554	0.450
16. Additional analyses^†^	39 (64)	0.900	0.473
17. Study selection	29 (26)	0.002^b.1^	0.297
18. Study characteristics	99 (88)	0.936	0.039^2^
19. Risk of bias within studies^‡^	63 (57)	0.989	0.006^b.2^
20. Results of individual studies	96 (86)	0.357	0.015^2^
21. Synthesis of results	110 (98)	0.657	0.997^b^
22. Risk of bias across studies^	29 (27)	0.791	0.797
23. Additional analysis˟	32 (55)	0.666^b^	0.706^b^
24. Summary of evidence	110 (98)	0.872^b^	0.997
25. Limitations	96 (86)	0.540	0.991
26. Conclusions	84 (75)	0.406	0.611
27. Funding	87 (78)	0.137	< 0.001^2^

*NA* not applicable, *PRISMA* preferred reporting items for systematic reviews and meta-analysis

^†^51 missing cases

^‡^1 missing case

^^^4 missing cases

˟54 missing cases

^1^Articles with no PRISMA mention had better reporting

^2^Articles published in journal requiring PRISMA adherence had better reporting

^a^Cochran-Mantel-Haenzal test used with controlling for type of study (systematic review or meta-analysis)

^b^Logistic regression used with controlling for type of study if Breslow-Day had *p*-value > 0.05

### Factors associated with better PRISMA adherence

The reviews mentioning the use of the PRISMA statement reported a mean of 18.6 (SE 0.4) PRISMA items adequately, while for the 45 reviews without mention of adherence to the PRISMA statement this was a mean of 17.8 (SE 0.5) items (*p*-value = 0.214). Comparison of each individual scoring item, between reviews with explicit mention of PRISMA adherence and without, showed only a significant difference in the results of study selection in favor of reviews with no mention of PRISMA adherence (*p*-value = 0.002).

Reviews published in journals that require authors to write their review in adherence to the PRISMA statement had better mean overall compliance to the statement (mean 19.2, SE 0.4) than reviews published in journals without such recommendations or requirements (mean 17.2, SE 0.5; *p*-value = 0.001). Journals requiring PRISMA adherence resulted in better reporting of the methods of study selection (*p*-value = 0.020), methods of risk of bias assessment within studies (*p*-value = 0.027), the study characteristics (*p*-value = 0.039), results of risk assessment within studies (*p*-value = 0.006), results of the individual studies (*p*-value = 0.015) and the funding sources (*p*-value < 0.001).

Reviews which only included RCTs in their analysis did not report more items adequately (mean 18.2, SE 0.8) compared to reviews included all sorts of reviews (mean 18.8, SE 0.3; *p* = 0.486). Furthermore, reviews with an author affiliated to a department of epidemiology and/or statistics did not report more items adequately (mean 19.9, SE 0.9) compared to reviews without authors who have such a background (mean 18.1, SE 0.3; *p-*value = 0.057).

## Discussion

The quality of reporting of the SRs and MAs published in the top five journals related to emergency medicine in 2015 and 2016 should be improved, as none of the reviews reported adequately on all items of the PRISMA statement. In this current study, reviews that were claimed to be written in adherence to the PRISMA statement did not show better overall reporting. However, reviews published in journals with the requirement to adhere to the PRISMA statement in their instructions to authors had a significant better quality of reporting, thereby providing the most robust results and contribute most to the evidence-based decision-making in the field of the emergency medicine.

Studies on the quality of reporting in other medical specialties showed comparable results: overall adherence to the PRISMA statement in fields such as orthodontic, surgery, and radiology have reported a mean of 17 up to 22 adequately adhered items [15–17]. Earlier studies reported inconclusive results regarding the positive association between claims of adhering to the PRISMA statement and quality of reporting [13, 15, 17, 18]. Our results are in line with the results reported within the surgical field by Adie et al., in which no evident association was found between reviews with and without a clear acknowledgement of the PRISMA statement [16]. However, journals requiring authors to explicitly mention adherence to a specific reporting guideline in their manuscript might enhance awareness for transparent reporting [13, 17, 19, 20]. The PRISMA statement is known to mainly focus on the reporting of reviews evaluating randomized trails, therefore, we assessed if reviews which only included RCT’s had better quality of reporting [5, 11]. The current study showed that only including RCT’s in the review did not influence the quality of reporting. A previous study assessed the association between the quality of reporting in articles with and without an author from a department of epidemiology or statistics, like this current study, they were unable to find an association [16].

Several of our findings about individual items of the PRISMA statement require extra attention. First, only 19% of the reviews reported on the existence of a review protocol and, if so, where it could be found. The use of a protocol can reduce duplication and outcome reporting bias, and contribute to an increase in research integrity, accountability and transparency [16, 21–26]. Therefore, the PRISMA statement recommends designing a protocol for MAs and requires authors to state if, or if not, a protocol was designed [11]. Apparently, authors are still hesitant to report not adhering to a protocol. Second, only 27% of the reviews reported an assessment of potential publication bias, despite it being known to be wide-spread within medical research [25, 27]. The risk of publication bias within SRs and MAs is highest during the selection process, resulting in the need of a transparent selection process to decrease this risk [28]. Our results showed that the reporting of the selection process is insufficiently done by most reviews, only 26% reported this adequately. Most reviews provided a flow diagram but were unable to adequately report reasons for exclusion at each stage of the study selection process, especially during the screening of titles and abstracts. Surprisingly, the reviews mentioning adherence to the PRISMA statement reported worse on this item compared to the reviews without explicit PRISMA adherence. The low scores on reporting of a protocol and the intention and results of risk of bias assessment are in line with earlier published studies [17, 20, 22].

The current study found that journals which require authors to adhere to the PRISMA statement for SRs and MAs published reviews with a statistically significant higher quality of reporting. The endorsement of reporting guidelines by medical journals has increased over the last couple of years, but these rates are still far from ideal [29, 30]. It has been nine years since the publication of the PRISMA statement and journal endorsement of reporting guidelines often stays behind, which does not differ within the field of emergency medicine [5, 24, 31, 32]. Tunis et al. found an increase in quality of reporting within two and a half year after implementation from 20.9 to 22.6 adequate reported items and Liu et al. found that reporting of item 1, 2, 12, 17 and 22 significantly improved after implementation of the PRISMA statement [17, 22]. Such substantial improvements in reporting were also seen within a couple of years after implementation of other reporting guidelines (e.g. STARD, CONSORT) [10, 31]. Therefore, to further improve the quality of reporting, journals should revise the instruction to authors by including the requirement, or at least a recommendation, to use a reporting guideline for the reporting of the submitted manuscript. This should be actively implemented by editorial teams and/or peer reviewers [24]. Mandating submission of a completed reporting guideline checklist might further increase quality of reporting, forcing authors to critically look at their quality of reporting before submission of the manuscript [10].

Other requirements made by journals could, on the contrary, negatively affect the quality of reporting in a variety of ways. It can be argued that a limited word count decreases authors’ ability to clearly report all items, and result in a suboptimal overall quality of reporting [16]. However, study results regarding the negative association between manuscript length and quality of reporting are inconclusive [16, 19].

The results of the current study should be interpreted in the right contact, since this study did not assess the methodological quality since the PRISMA statement is only meant to assess quality of reporting. For assessment of methodological quality are other validated tools available, such as the AMSTAR tool (A MeaSurement Tool to Assess systematic Reviews) [33]. Use of the PRISMA statement does not negatively influence the methodological quality. Nonetheless, previous conducted studies have found that adherence to the PRISMA statement improves the methodological quality of studies and vice versa [13, 14, 17]. Thereby, helping authors to write clear, complete and transparent reviews to improve the quality of reporting of SRs and MAs.

## Conclusion

The current quality of reporting of SRs and MAs within the top five journals of emergency medicine related literature is could be improved, nonetheless is comparable to other medical specialties. There was no statistically significant difference between reviews explicitly stating the use of the PRISMA statement and reviews without stating adherence to the PRISMA statement. Reviews from journals which require adherence to a reporting guideline had a higher quality of reporting than reviews from journals which did not endorse. Since the limited availability of SRs and MAs in the field of emergency medicine, authors should use a reporting guideline such as the PRISMA statement to improve the quality of reporting of their reviews and thereby increasing awareness and transparency of both reporting and methodological quality of SRs and MAs.

## Additional files

Additional file 1: PRISMA statement assessment criteria. Full pre-specified list of criteria used to assess each individual item of the PRISMA statement for all included reviews. ^1^*Not applicable* was assigned if it concerned a systematic review, since additional analyses is only applicable to meta-analyses. ^2^*Not applicable* was assigned if it was mentioned earlier that no risk of bias assessment would be performed. ^3^*Not applicable* was assigned in case of a meta-analysis if it was previously mentioned no additional analyses would be performed. (PDF 47 kb)

Additional file 2: Baseline characteristics of the included reviews. Complete baseline characteristics given of all included reviews. *AcEM* = Academic of Emergency Medicine, *AnEM =* Annals of Emergency Medicine, *SJTREM* = Scandinavian Journal of Trauma, Resuscitation and Emergency Medicine. *Full reference can be found in Additional file 3. (PDF 166 kb)

Additional file 3: Reference list of the included reviews. (PDF 147 kb)

Additional file 4: PRISMA assessment of each individual review. Final scores for all reviews based on each individual idem of the PRISMA statement. *Full references can be found in Additional file 3. (PDF 976 kb)

## Abbreviations

5-YIF: 5-Years impact factor
AMSTAR: A measurement tool to assess systematic reviews
CONSORT: Consolidated standards of reporting trials
IQR: Interquartile range
MA: Meta-analysis
PRISMA: Preferred reporting items for systematic reviews and meta-analyses
RCT: Randomized control trail
SD: Standard deviation
SE: Standard error
SR: Systematic review
STARD: Standard for the reporting of diagnostic accuracy studies
