# Supplementary material for: Quality of reporting of systematic reviews and meta-analyses in emergency medicine based on the PRISMA statement
Source: BMC Emerg Med. 2019 Feb 11;19:19. doi: 10.1186/s12873-019-0233-6 (PMC6371507; doi:10.1186/s12873-019-0233-6)
Supplement: Supplementary file 1 — PRISMA statement assessment criteria. Full pre-specified list of criteria used to assess each individual item of the PRISMA statement for all included reviews. 1Not applicable was assigned if it concerned a systematic review, since additional analyses is only applicable to meta-analyses. 2Not applicable was assigned if it was mentioned earlier that no risk of bias assessment would be performed. 3Not applicable was assigned in case of a meta-analysis if it was previously mentioned no additional analyses would be performed. (PDF 47 kb) [file 12873_2019_233_MOESM1_ESM.pdf]

PRISMA statement assessment criteria

Full prespecified list of criteria used to assess each individual item of the PRISMA statement for every included article

Section/topic	#	Checklist item
TITLE		
Title	1	Identifies the report as a systematic review and/or meta-analysis. If meta-analysis is performed it must be mentioned.
ABSTRACT		
Structured summary	2	Provides a structured summary with subheadings.
INTRODUCTION		
Rationale	3	Describes the rationale for the review in the context of what is already known.
Objectives	4	Provides an explicit statement of questions being addressed with as much reference as possible to participants, interventions, comparisons and outcomes (PICO).
METHODS		
Protocol and registration	5	Reports if a review protocol exists. When a protocol exists, it should be indicated where it can be accessed (e.g. unpublished, upon request or reference to published protocol article, web address or registration database).
Eligibility criteria	6	Specifies both study characteristics (e.g. study design, length of follow-up, PICOS) and report characteristics (e.g. years considered, language, publication status) used as criteria for inclusion and/or exclusion of articles.
Information sources	7	Describes all information sources used and date (or month) last searched.
Search	8	Presents a full electronic search strategy for at least one database, including if or which limits are used within the search, such that it could be repeated.
Study selection	9	States the process for selecting studies (e.g. blinded, how consensus was reached in case of disagreement), including if this is done independently or in duplicate.
Data collection process	10	Describes the methods of data extraction from included studies (e.g. piloted forms, process for obtaining data, contact authors from included articles), including if data was extracted independently or in duplicate.
Data items	11	Lists and defines all variables for which data were sought (e.g. PICO).
Risk of bias in individual studies	12	Describes methods used for assessing risk of bias (or quality assessment) of individual studies. If no risk assessment was done, it should be stated why.
Summary measures	13	States the principal summary measure used (e.g. risk ratio, difference in means).
Synthesis of results	14	In case of a systematic review, the methods of handling data were reported or reasons why meta-analysis was not performed.<br>In case of a meta-analysis, the methods used to combine the results were reported (e.g. pooled measures), including measures of consistency (such as I^2) and/or heterogeneity assessment.
Risk of bias across studies	15	Describes the methods used for assessing risk of publication bias across the studies of the systematic review (e.g. funnel plot, eggers test, begs test). If no risk of publication bias assessment was done, it should be stated why.
Additional analyses[1]	16	In case of a meta-analysis, the methods of additional analyses are reported (e.g. sensitivity analyses, subgroup analyses, meta-regression).
RESULTS		

Study selection	17	Gives the number of screened studies, assessed for eligibility, included in the review, and if applicable included in the meta-analysis, with reason for exclusion at each stage(e.g. using a PRISMA flow diagram). A detailed reasoning for exclusion after full-text review must be present.
Study characteristics	18	For each included study, both characteristics for which data were extracted (e.g. study size, PICOS, follow-up period) and references for included studies are given.
Risk of bias within studies[2]	19	Presents detailed result of the risk of bias assessment (or quality assessment) for each study.
Results of individual studies	20	Reports the results of all considered outcomes for each study (e.g. using a table or a forest plot).<br>In case of a meta-analysis, the effect estimates and confidence intervals are also provided.
Synthesis of results	21	Reports the main results (overall results of considered outcomes). In case of a meta-analysis, confidence intervals and measures of consistency were presented.
Risk of bias across studies[2]	22	Presents results of risk of publication bias assessment across studies (e.g. funnel plot). If eventually no assessment of publication bias could be performed, it should be stated why.
Additional analysis[3]	23	In case of a meta-analysis, the results of additional analyses were presented including estimates, effect sizes and confidence intervals. If none could be done, it should be stated why.
DISCUSSION		
Summary of evidence	24	Summarizes the main findings and discusses the applicability of the finding.
Limitations	25	Discusses limitations of study (e.g. risk of bias, incomplete retrieval of identified research, reporting bias).
Conclusions	26	Provides a general interpretation of the results and gives implications for future research.
FUNDING		
Funding	27	Describes sources of funding for the systematic review.

[1]*Not applicable* was assigned if it concerned a systematic review, since additional analyses is only applicable to meta-analyses. [2]*Not applicable* was assigned if it was mentioned earlier that no risk of bias assessment would be performed. [3]*Not applicable* was assigned in case of a meta-analysis when it was previously mentioned no additional analyses would be performed.
