# Supplementary material for: Quality of reporting of systematic reviews and meta-analyses in emergency medicine based on the PRISMA statement
Source: BMC Emerg Med. 2019 Feb 11;19:19. doi: 10.1186/s12873-019-0233-6 (PMC6371507; doi:10.1186/s12873-019-0233-6)
Supplement: Supplementary file 2 — Baseline characteristics of the included reviews. Complete baseline characteristics given of all included reviews. AcEM = Academic of Emergency Medicine, AnEM = Annals of Emergency Medicine, SJTREM = Scandinavian Journal of Trauma, Resuscitation and Emergency Medicine. *Full reference can be found in Additional file 3. (PDF 166 kb) [file 12873_2019_233_MOESM2_ESM.pdf]

Baseline characteristics of the included reviews

Article*	Year	Journal	Number of authors	Author with affiliation to department of epidemiology or statistics	Country of origin (based on first author)	Systematic review or meta-analysis	Number of articles included in systematic review or qualitative analysis	PRISMA use mentioned
**Ali**[1]	2015	Injury	2	No	United Kingdom	Systematic review	28	Yes
**Asha**[2]	2015	AnEM	2	No	Australia	Meta-analysis	5	No
**Aurégan** [3]	2015	Injury	2	No	France	Systematic review	3	Yes
**Aurégan** [4]	2015	Injury	5	No	France	Systematic review	12	Yes
**Bakhshayesh** [5]	2016	SJTREM	3	No	Sweden	Systematic review	16	Yes
**Barquet** [6]	2015	Injury	3	No	Uruguay	Systematic review	160	Yes
**Bartlett** [7]	2016	Injury	3	No	United Kingdom	Systematic review	52	No
**Bellollio** [8]	2016	AcEM	8	No	USA	Meta-analysis	55	Yes
**Benoit** [9]	2015	Resuscitation	4	No	USA	Meta-analysis	10	No
**Bhate** [10]	2015	Resuscitation	4	Yes	Canada	Systematic review	9	Yes
**Bonnes** [11]	2016	AnEM	7	No	The Netherlands	Meta-analysis	20	Yes
**Bougouin** [12]	2015	Resuscitation	12	No	France	Meta-analysis	13	No
**Buis** [13]	2016	Resuscitation	5	No	The Netherlands	Systematic review	13	Yes
**Camarda** [14]	2016	Injury	5	No	Italy	Systematic review	9	Yes
**Carpenter** [15]	2016	AcEM	7	No	USA	Meta-analysis	34	Yes
**Carpenter** [16]	2015	AcEM	7	No	USA	Meta-analysis	34	Yes
**Cartledge** [17]	2016	Resuscitation	5	Yes	Australia	Systematic review	26	Yes
**Chaikriangkrai** [18]	2016	AnEM	8	No	USA	Meta-analysis	8	No
**Chao** [19]	2016	AcEM	6	No	USA	Meta-analysis	5	No
**Chapman** [20]	2016	Resuscitation	4	No	United Kingdom	Meta-analysis	55	No
**Cheng** [21]	2015	Resuscitation	6	No	Canada	Meta-analysis	14	Yes
**Chopra** [22]	2016	Resuscitation	4	No	Canada	Meta-analysis	6	Yes
**Chou** [23]	2015	Resuscitation	8	No	Taiwan	Meta-analysis	12	Yes
**Cohen** [24]	2015	AnEM	6	Yes	Canada	Systematic review	10	No
**Couper** [25]	2016	Resuscitation	6	No	Taiwan	Meta-analysis	9	No
**Cournoyer** [26]	2016	AcEM	6	No	Canada	Meta-analysis	20	Yes
**Davis** [27]	2015	AcEM	6	No	USA	Meta-analysis	17	No
**Dekker** [28]	2016	Injury	3	No	The Netherlands	Meta-analysis	13	Yes
**Descatha** [29]	2015	Resuscitation	6	Yes	France	Meta-analysis	17	Yes
**Deslarzes** [30]	2016	SJTREM	5	No	Switzerland	Meta-analysis	183	No

**Doleman** [31]	2015	Injury	2	No	United Kingdom	Meta-analysis	12	No
**Edwards** [32]	2016	Injury	5	No	United Kingdom	Meta-analysis	41	Yes
**Ekmejian** [33]	2016	SJTREM	4	No	Australia	Systematic review	2	Yes
**Eliyahu** [34]	2016	AcEM	4	No	Canada	Systematic review	5	Yes
**Galipeau** [35]	2015	AcEM	10	No	Canada	Meta-analysis	10	Yes
**Garara** [36]	2016	Injury	6	No	United Kingdom	Systematic review	12	Yes
**Gates** [37]	2015	Resuscitation	6	No	United Kingdom	Meta-analysis	5	No
**Ghayoumi** [38]	2015	Injury	3	No	USA	Meta-analysis	21	No
**Guest** [39]	2016	Injury	5	No	Australia	Systematic review	12	No
**Hajibandeh** [40]	2015	Injury	3	No	United Kingdom	Meta-analysis	7	Yes
**Harmsen** [41]	2015	Injury	6	No	The Netherlands	Systematic review	20	Yes
**Hartling** [42]	2016	AcEM	7	No	Canada	Systematic review	14	No
**Hsieh** [43]	2016	Resuscitation	6	Yes	Taiwan	Systematic review	22	Yes
**Huang** [44]	2015	Resuscitation	13	No	China	Meta-analysis	8	Yes
**Hyldmo** [45]	2015	SJTREM	7	No	Norway	Systematic review	13	Yes
**Hyldmo** [46]	2015	SJTREM	7	No	Norway	Meta-analysis	43	Yes
**Inácio** [47]	2016	Resuscitation	7	No	Brazil	Meta-analysis	23	Yes
**Inge** [48]	2016	Injury	4	No	The Netherlands	Systematic review	10	No
**Jiang** [49]	2016	SJTREM	4	No	China	Meta-analysis	6	Yes
**Juurlink** [50]	2015	AnEM	7	No	Canada	Systematic review	84	No
**Kim** [51]	2016	Resuscitation	5	No	Korea	Meta-analysis	14	No
**Laan** [52]	2016	Injury	7	No	USA	Meta-analysis	13	Yes
**Leonard**[53]	2015	SJTREM	7	No	USA	Systematic review	15	Yes
**Li** [54]	2016	SJTREM	5	No	China	Meta-analysis	12	No
**Martindale** [55]	2016	AcEM	9	No	USA	Meta-analysis	57	Yes
**Marufu** [56]	2015	Injury	3	No	United Kingdom	Systematic review	30	Yes
**McCarthy** [57]	2016	Injury	3	No	Australia	Systematic review	59	No
**McMahon** [58]	2016	Injury	5	No	United Kingdom	Systematic review	13	Yes
**McQueen** [59]	2015	Injury	4	No	United Kingdom	Systematic review	16	Yes
**Menon** [60]	2016	AcEM	7	No	USA	Systematic review	31	No
**Mikalsen** [61]	2016	SJTREM	3	No	Norway	Systematic review	26	No
**Millin** [62]	2016	Resuscitation	9	No	USA	Meta-analysis	12	Yes
**Mockford** [63]	2015	Resuscitation	8	No	United Kingdom	Systematic review	47	No
**Morelli** [64]	2016	Injury	6	No	Italy	Meta-analysis	17	Yes
**Nunn** [65]	2016	Injury	3	No	Canada	Systematic review	12	Yes
**Nyholm** [66]	2016	Injury	5	No	Denmark	Systematic review	30	No

**O'Dochartaigh** [67]	2015	Injury	2	No	Canada	Systematic review	8	Yes
**Olaussen** [68]	2015	Resuscitation	6	Yes	Australia	Systematic review	9	Yes
**Ortega-Deballon**[69]	2016	Resuscitation	5	No	Canada	Systematic review	21	No
**Oteir** [70]	2015	Injury	5	Yes	Australia	Systematic review	8	No
**Oto** [71]	2015	AcEM	5	No	USA	Systematic review	12	No
**Papakostidis** [72]	2015	Injury	4	No	Greece	Meta-analysis	7	Yes
**Patel** [73]	2016	Resuscitation	2	No	USA	Systematic review	9	Yes
**Peeters** [74]	2016	Injury	6	No	The Netherlands	Systematic review	49	No
**Peters** [75]	2015	Injury	6	No	The Netherlands	Systematic review	36	No
**Quan** [76]	2016	Resuscitation	6	Yes	USA	Systematic review	24	No
**Ramaekers** [77]	2016	AcEM	4	Yes	Canada	Meta-analysis	15	No
**Raven** [78]	2016	AnEM	5	Yes	USA	Systematic review	13	Yes
**Reynold** [79]	2015	Resuscitation	5	No	USA	Meta-analysis	9	Yes
**Roberts** [80]	2015	Resuscitation	6	No	USA	Systematic review	17	No
**Ruff** [81]	2015	Injury	5	No	USA	Systematic review	11	No
**Sammy** [82]	2016	Injury	5	No	United Kingdom	Meta-analysis	15	Yes
**Sanfilippo** [83]	2016	Resuscitation	7	No	Italy	Meta-analysis	7	Yes
**Schenone** [84]	2016	Resuscitation	8	No	USA	Meta-analysis	24	Yes
**Schimmer** [85]	2016	Injury	3	No	The Netherlands	Systematic review	12	No
**Sharples** [86]	2016	Injury	2	No	United Kingdom	Meta-analysis	7	Yes
**Sharrock** [87]	2016	Injury	5	No	United Kingdom	Meta-analysis	26	Yes
**Shen** [88]	2015	Injury	9	No	China	Meta-analysis	10	Yes
**Shopp** [89]	2015	AcEM	4	No	USA	Meta-analysis	10	Yes
**Sin** [90]	2015	AcEM	3	No	USA	Systematic review	4	No
**Sin** [91]	2016	AcEM	4	No	USA	Systematic review	14	Yes
**Slessor** [92]	2015	AnEM	2	No	United Kingdom	Meta-analysis	27	No
**Slobogean** [93]	2015	Injury	3	Yes	Canada	Meta-analysis	41	No
**Smith** [94]	2015	Resuscitation	2	No	United Kingdom	Systematic review	9	Yes
**Spanos** [95]	2016	Injury	4	No	Greece	Systematic review	23	No
**Stang** [96]	2015	AcEM	5	No	Canada	Systematic review	32	Yes
**Strudwick** [97]	2015	AcEM	6	No	Australia	Systematic review	15	Yes
**Subramaniam** [98]	2016	AcEM	4	No	USA	Meta-analysis	6	Yes
**Talikowska** [99]	2015	Resuscitation	3	No	Australia	Meta-analysis	22	No
**Unsworth** [100]	2015	SJTREM	3	No	Australia	Systematic review	40	No
**Vargas** [101]	2015	Resuscitation	6	No	Italy	Meta-analysis	6	Yes
**Veigas** [102]	2016	SJTREM	5	No	Canada	Systematic review	13	Yes

**Villa-Roel [103]**	2016	AcEM	6	No	Canada	Meta-analysis	5	Yes
**Vrablik [104]**	2015	AnEM	6	No	USA	Meta-analysis	3	No
**Wang [105]**	2016	AnEM	6	Yes	USA	Meta-analysis	8	Yes
**West [106]**	2016	Resuscitation	3	No	United Kingdom	Systematic review	22	Yes
**Williams [107]**	2016	Resuscitation	5	Yes	Australia	Meta-analysis	8	Yes
**Yan [108]**	2015	AcEM	3	No	Canada	Meta-analysis	6	No
**Young [109]**	2016	AnEM	2	No	USA	Systematic review	80	No
**Zhang [110]**	2015	SJTREM	5	No	China	Meta-analysis	10	Yes
**Zhang [111]**	2015	SJTREM	6	No	China	Meta-analysis	14	Yes
**Zhao [112]**	2015	Resuscitation	6	No	China	Meta-analysis	4	No

AcEM = Academic of Emergency Medicine; AnEM = Annals of Emergency Medicine; SJTREM = Scandinavian Journal of Trauma, Resuscitation and Emergency Medicine.
*Full reference can be found in Appendix file 3.
