# Supplementary material for: Quality of reporting of systematic reviews and meta-analyses in emergency medicine based on the PRISMA statement
Source: BMC Emerg Med. 2019 Feb 11;19:19. doi: 10.1186/s12873-019-0233-6 (PMC6371507; doi:10.1186/s12873-019-0233-6)
Supplement: Supplementary file 3 — Reference list of the included reviews. (PDF 147 kb) [file 12873_2019_233_MOESM3_ESM.pdf]

Reference list of the included reviews

1. Ali AM, Willett K. What is the effect of the weather on trauma workload? A systematic review of the literature. Injury. 2015;46:945-953
2. Asha SE, Miers JW. A systematic review and meta-analysis of D-dimer as a rule-out test for suspected acute aortic dissection. Annals of Emergency Medicine. 2015; 66(4):368-378
3. Aurégan JC, Bégué T. Bioactive glass for long bone infection: a systematic review. Injury. 2015;46 S8:S3-S7
4. Aurégan JC, Bégué T, Rigoulot G, et al. Success rate and risk factors of failure of the induced membrane technique in children: a systematic review. Injury. 2016;S62-S67
5. Bakhshayesh P, Boutefnouchet T, Tötterman A. Effectiveness of non invasive external pelvic compression: a systematic review of the literature. Scandinavian Journal of Trauma, Resuscitation and Emergency Medicine. 2016;24:73
6. Barquet A, Gelink A, Giannoudis PV. Proximal femoral fractures and vascular injuries in adults: incidence, aetiology and outcome. Injury. 2015;46:2297-2313
7. Bartlett A, Williams R, Hilton M. Splenic rupture in infectious mononucleosis: a systematic review of published case reports. Injury. 2016;531-538
8. Bellolio MF, Gilani WI, Barrionuevo P, et al. Incidence of adverse events in adults undergoing procedural sedation in the emergency department: a systematic review and meta-analysis. Academic Emergency Medicine. 2016;23:119:134
9. Benoit JL, Gerecht RB, Steuerwald MT, et al. Endotracheal intubation versus supraglottic airway placement in out-of-hospital cardiac arrest: a meta-analysis. Resuscitation. 2015; 93: 20-26
10. Bhate TD, McDonald B, Sekhon MS, et al. Association between blood pressure and outcome in patients after cardiac arrest: a systematic review. Resuscitation. 2015; 97:1-6
11. Bonnes JL, Brouwer MA, Navarese EP, et al. Manual cardiopulmonary resuscitation versus CPR including a mechanical chest compression device in out-of-hospital cardiac arrest: a comprehensive meta-analysis from randomized and observational studies. Annals of Emergency Medicine. 2016; 67(3):349-360
12. Bougouin W, Mustafic H, Marijon E, et al. Gender and survival after sudden cardiac arrest: a systematic review and meta-analysis. Resuscitation. 2015; 94:55-60
13. Buis ML, Maissan IM, Hoeks SE, et al. Defining the learning curve for endotracheal intubation using direct laryngoscopy: a systematic review. Resuscitation. 2016;90:63-71
14. Camarda L, Morello S, Balistreri F, et al. Non-metallic implant for patellar fracture fixation: a systematic review. Injury. 2016;47:1613-1617
15. Carpenter CR, Hussain AM, Ward MJ, et al. Spontaneous subarachnoid hemorrhage: a systematic review and meta-analysis describing the diagnostic accuracy of history, physical examination, imaging, and lumbar puncture with an exploration of test thresholds. Academic Emergency Medicine. 2016;23:963-1003
16. Carpenter CR, Shelton E, Fowler S, et al. Risk factors and screening instruments to predict adverse outcomes for undifferentiated older emergency department patients: a systematic review and meta-analysis. Academic Emergency Medicine. 2015;22:1-21
17. Cartledge S, Bray JE, Leary M, et al. A systematic review of basic life support training targeted to family members of high-risk cardiac patients. Resuscitation. 2016;105:70-78
18. Chaikriangkrai K, Shantha GPS, Jhun HYJ, et al. Prognostic value of coronary artery calcium score in acute chest pain patients without know coronary artery disease: systematic review and meta-analysis. Annals of Emergency Medicine. 2016;68(6):659-670
19. Chao JH, Lin RCJ, Marneni S, et al. Predictors of airspace disease on chest X-ray in emergency department patients with clinical bronchiolitis: a systematic review and meta-analysis. Academic Emergency Medicine. 2016;23:1107-1118
20. Chapman SM, Wray J, Oulton K, et al. Systematic review of paediatric track and trigger systems for hospitalized children. Resuscitation. 2016;109:87-109

21. Cheng A, Lockey A, Bhanji F, et al. The use of high-fidelity manikins for advanced life support training – A systematic review and meta-analysis. Resuscitation. 2015; 93:142-149
22. Chopra AS, Wong N, Ziegler CP, et al Systematic review and meta-analysis of hemodynamic-directed feedback during cardiopulmonary resuscitation in cardiac arrest. Resuscitation. 2016;101:102-107
23. Chou EH, Dickman E, Tsou PY, et al. Ultrasonography for confirmation of endotracheal tube placement: a systematic review and meta-analysis. Resuscitation. 2015;90:97-103
24. Cohen L, Athaide V, Wickham ME, et al. The effect of ketamine of intracranial and cerebral perfusion pressure and health outcomes: a systematic review. Annals of Emergency Medicine. 2015;65(1):43-51
25. Couper K, Yeung J, Nicholson T, et al. Mechanical chest compression devices at in-hospital cardiac arrest: a systematic review and meta-analysis. Resuscitation. 2016;103:24-31
26. Cournoyer A, Iseppon M, Chauny JM, et al. Near-infrared spectroscopy monitoring during cardiac arrest: a systematic review and meta-analysis. Academic Emergency Medicine. 2016;23:851-862
27. Davis J, Czerniski B, Au A, et al. Diagnostic accuracy of ultrasonography in retained soft tissue foreign bodies: a systematic review and meta-analysis. Academic Emergency Medicine. 2015;22:777-787
28. Dekker AE, Krijnen P, Schipper IB. Results of crossed versus lateral entry K-wire fixation of displaced pediatric supracondylar humeral fractures: a systematic review and meta-analysis. Injury. 2016;47:2391-2398
29. Descatha A, Dagrenat C, Cassan P, et al. Cardiac arrest in the workplace and its outcome: a systematic review and meta-analysis. Resuscitation. 2015;96:30-36
30. Deslarzes T, Rousson V, Yersin B, et al. An evaluation of the Swiss staging model for hypothermia using case reports from the literature. Scandinavian Journal of Trauma, Resuscitation and Emergency Medicine. 2016;24:16
31. Doleman B, Moppett IK. Is early fracture surgery safe for patients on clopidogrel? Systematic review, meta-analysis and meta regression. Injury. 2015;46:954-962
32. Edwards DS, McMenemy L, Stapley SA, et al. 40 years of terrorist bombings – a meta-analysis of the casualty and injury profile. Injury. 2016;47:646-652
33. Ekmejian R, Sarrami P, Naylor JM, et al. A systematic review on the effectiveness of back protectors for motorcyclist. Scandinavian Journal of Trauma, Resuscitation and Emergency Medicine. 2016;24:115
34. Eliyahu L, Kirkland S, Campbell S, et al. The effectiveness of early educational interventions in the emergency department to reduce incidence of severity of postconcussion syndrome following a concussion: a systematic review. Academic Emergency Medicine. 2016;23:531-542
35. Galipeau J, Pussegoda K, Stevens A, et al. Effectiveness and safety of short-stay units in the emergency medicine department: a systematic review. Academic Emergency Medicine. 2015;22:893-907
36. Garara B, Wood A, Marcus HJ, et al. Intramuscular diaphragmatic stimulation for patients with traumatic high cervical injuries and ventilator dependent respiratory failure: a systematic review of safety and effectiveness. Injury;2016:539-544
37. Gates S, Quinn T, Deakin CD, et al. Mechanical chest compression for out of hospital cardiac arrest: systematic review and meta-analysis. Resuscitation. 2015;94:91-97
38. Ghayoumi P, Kandemir U, Morshed S. Evidence based update: open versus closed reduction. Injury. 2015;46:467-473
39. Guest R, Tran Y, Gopinath B, et al. Psychological distress following a motor vehicle crash: a systematic review of preventative interventions. Injury; 2016:2415-2423
40. Hajibandeh S, Hajibandeh S, Idehen N. Meta-analysis of the effect of tertiary survey on missed injury rate in trauma patients. Injury. 2015;46:2474-2482

41. Harmsen AMK, Giannakopoulos GF, Moerbeek PR, et al. The influence of prehospital time of trauma patients outcome: a systematic review. Injury. 2015; 46:602-609
42. Hartling L, Milne A, Foisy M, et al. What works and what's safe in pediatric emergency procedural sedation: an overview of reviews. Academic Emergency Medicine. 2016;23:519-530
43. Hsieh MJ, Bhanji F, Chiang WC, et al. Comparing the effects of self-instruction with that of traditional instruction in basic life support courses – A systematic review. Resuscitation. 2016;108:8-19
44. Huang FY, Huang BT, Wang PJ, et al. The efficacy and safety of prehospital therapeutic hypothermia in patients with out-of-hospital cardiac arrest: a systematic review and meta-analysis. Resuscitation. 2015;96:170-179
45. Hyldmo PK, Vist GE, Feyling AC, et al. Does turning trauma patients with an unstable spinal injury from the supine to a lateral position increase the risk of neurological deterioration? – a systematic review. Scandinavian Journal of Trauma, Resuscitation and Emergency Medicine. 2015;23:65
46. Hyldmo PK, Vist GU, Feyling AC, et al. Is the suspine position associated with loss of airway patency in unconscious trauma patients? A systematic review and meta-analysis. Scandinavian Journal of Trauma, Resuscitation and Emergency Medicine. 2015;23:50
47. Inácio JFS, da Rosa MSG, Shah J, et al. Monophasic and biphasic shock for transthoracic conversion of atrial fibrillation: systematic review and network meta-analysis. Resuscitation. 2016;100:66-75
48. Inge SY, Pull ter Gunne AF, Aarts CAM, et al. A systematic review of dynamic versus static distal tibofibular fixation. Injury. 2016;2627-2634
49. Jiang LB, Zhang M, Jiang SY, et al. Early goal-directed resuscitation for patients with severe sepsis and septic shock: a meta-analysis and trial sequential analysis. Scandinavian Journal of Trauma, Resuscitation and Emergency Medicine. 2016;24:23
50. Juurlink DN, Gosselin S, Kielstein JT, et al. Extracorporeal treatment for salicylate poisoning: systematic review and recommendations from the EXTRIP workgroup. Annals of Emergency Medicine. 2015;66(2):165-181
51. Kim SJ, Kim HJ, Lee HY, et al. Comparing extracorporeal cardiopulmonary resuscitation with conventional cardiopulmonary resuscitation: a meta-analysis. Resuscitation. 2016;103:106-116
52. Laan DV, Vu TDN, Thiels CA, et al. Chest wall thickness and decompression failure: a systematic review and meta-analysis comparing anatomic locations in needle thoracostomy. Injury. 2016;47:797-804
53. Leonard J, Garrett RE, Salottolo K, et al. Cerebral salt wasting after traumatic brain injury: a review of the literature. Scandinavian Journal of Trauma, Resuscitation and Emergency Medicine. 2015;23:98
54. Li H, Wang D, Yu Y, et al. Mechanical versus manual chest compressions for cardiac arrest: a systematic review and meta-analysis. Scandinavian Journal of Trauma, Resuscitation and Emergency Medicine. 2016;24:10
55. Martindale JL, Wakai A, Collins SP, et al. Diagnosing acute heart failure in the emergency department: a systematic review and meta-analysis. Academic Emergency Medicine. 2016;23:223-242
56. Marufu TC, Mannings A, Moppett IK. Risk scoring models for predicting peri-operative morbidity and mortality in people with fragility hip fractures: qualitative systematic review. Injury. 2015;46:2325-2334
57. McCarthy A, Curtis K, Holland AJA. Paediatric trauma systems and their impact on the health outcomes of severely injured children: an integrative review. Injury. 2016;47:574-584
58. McMahon SE, Little ZE, Smith TO, et al. The management of segmental tibial shaft fractures: a systematic review. Injury. 2016;47:568-573

59. McQueen C, Smyth M, Fisher J, et al. Does the use of dedicated dispatch criteria by Emergency Medical Servicse optimize appropriate allocation of advanced care resources in cases of high severity trauma? A systematic review. Injury. 2015;46:1197-1206
60. Menon AA, Nganga-Good C, Martis M, et al. Linkage-to-care methods and rates in U.S. emergency department-based HIV testing programs: a systematic literature review brief report. Academic Emergency Medicine. 2016;23:835-842
61. Mikalsen IB, Davis P, Øymar K. High flow nasal cannula in children: a literature review. Scandinavian Journal of Trauma, Resuscitation and Emergency Medicine. 2016;24:93
62. Millin MG, Comer AC, Nable JV, et al. Patients without ST elevation after return of spontaneous circulation may benefit from emergent percutaneous intervention: a systematic review and meta-analysis. Resuscitation. 2016;108:54-60
63. Mockford C, Fritz Z, George R, et al. Do not attempt cardiopulmonary resuscitation (DNACPR) orders: a systematic review of the barriers and facilitators of decision-making and implementation. Resuscitation. 2015;88:99-113
64. Morelli I, Drago L, George DA, et al. Masquelet technique: myth or reality? A systematic review and meta-analysis. Injury. 2016;S68-S76
65. Nunn J, Erdogan M, Green RS. The prevalence of alcohol-related trauma recidivism: a systematic review. Injury. 2016;47-551-558
66. Nyholm AM, Palm H, Malchau H, et al. Lacking evidence for performance of implants used for proximal femoral fractures – a systematic review. Injury 2016;47:586-594
67. O'Dochartaigh D, Douma M. Prehospital ultrasound of the abdomen and thorax changes trauma patient management: a systematic review. Injury. 2015;46:2093-2102
68. Olaussen A, Shepherd M, Nehme Z, et al. Return of consciousness during ongoing cardiopulmonary resuscitation: a systematic review. Resuscitation. 2015; 86:44-48
69. Ortega-Deballon I, Hornby L, Shemie SD, et al. Extracorporeal resuscitation for refractory out-of-hospital cardiac arrest in adults: a systematic review of international practices and outcomes. Resuscitation. 2016; 101:12-20
70. Oteir AO, Smith K, Stoelwinder JU, et al. Should suspected cervical spinal cord injury be immobilized? A systematic review. Injury. 2015;46:528-535
71. Oto B, Corey DJ, Oswald J, et al. Early secondary neurologic deterioration after blunt spinal trauma: a review of the literature. Academic Emergency Medicine. 2015; 22:1200-121
72. Papakostidis C, Panagiotopoulos A, Piccioli A, et al. Timing of internal fixation of femoral neck fractures. A systematic review and meta-analysis of the final outcome. Injury. 2015;46:459:466
73. Patel JK, Parikh PB. Association between therapeutic hypothermia and long-term quality of life in survivors of cardiac arrest: a systematic review. Resuscitation. 2016;103:54-59
74. Peeters CMM, Visser E, van de Ree CLP, et al. Quality of life after hip fracture in the elderly: a systematic review. Injury. 2016;47:1369-1382
75. Peters RM, Claessen MAP, Doornberg JN, et al. Union rate after operative treatment of humeral shaft nonunion – a systematic review. Injury. 2015;46:2314-2324
76. Quan L, Bierens JJLM, Lis R, et al. Predicting outcome of drowning at the scene: a systematic review and meta-analyses. Resuscitation. 2016;104:63-75
77. Ramaekers R, Mukarram M, Smith CAM, et al. The predictive value of preendoscopic risk scores to predict adverse outcomes in emergency department patients with upper gastrointestinal bleeding: a systematic review. Academic Emergency Medicine. 2016;23:1218-1227
78. Raven MC, Kushel M, Ko MJ, et al. The effectiveness of emergency department visit reduction programs: a systematic review. Annals of Emergency Medicine. 2016;68(4):467-483
79. Reynold JC, Raffay V, Lang E, et al. When should chest compressions be paused to analyse the cardiac rhythm? A systematic review and meta-analysis. Resuscitation. 2015;97:38-47

80. Roberts BW, Karagiannis P, Coletta M, et al. Effects of $PaCO_2$ derangements of clinical outcomes after cerebral injury: a systematic review. Resuscitation. 2015;91:32-41
81. Ruff J, Wang JL, Quatman-Yates CC, et al. Commercially available gaming systems as clinical assessment tools to improve value in the orthopaedic setting: a systematic review. Injury. 2015;46:178-183
82. Sammy I, Lecky F, Sutton A, et al. Factors affecting mortality in older trauma patients – a systematic review and meta-analysis. Injury. 2016;47:1170-1183
83. Sanfilippo F, Corredor C, Santonocito C, et al. Amiodarone or lidocaine for cardiac arrest: a systematic review and meta-analysis. Resuscitation. 2016;107:31-37
84. Schenone AL, Cohen A, Patarroyo G, et al. Therapeutic hypothermia after cardiac arrest: a systematic review/meta-analysis exploring the impact of expanded criteria and targeted temperature. Resuscitation. 2016;108:102-110
85. Schimmer JAG, van der Steeg AFW, Zuidema WP. Splenic function after angioembolization for splenic trauma in children and adults: a systematic review. Injury. 2016;47-525-530
86. Sharples A, Brohi K. Can clinical prediction tools predict the need for computed tomography in blunt abdominal? A systematic review. Injury. 2016;47:1811-1818
87. Sharrock AE, Barker T, Yuen HM, et al. Management and closure of the open abdomen after damage control laparotomy for trauma. A systematic review and meta-analysis. Injury. 2016;47:296-306
88. Shen GS, Li Y, Zhao GY, Zhou GY, et al. Cigarette smoking and risk of hip fracture in women: a meta-analysis of prospective cohort studies. Injury. 2015; 46:1333-1340
89. Shopp JD, Stewart LK, Emmett TW, et al. Findings from 12-lead electrocardiography that predict circulatory shock from pulmonary embolism: systematic review and meta-analysis. Academic Emergency Medicine. 2015;22:1127-1137
90. Sin B, Ternas T, Motov SM. The use of subdissociative-dose ketamine for acute pain in the emergency department. Academic Emergency Medicine. 2015;22:251-257
91. Sin B, Wai M, Tatunchak T, et al. The use of intravenous acetaminophen for acute pain in the emergency department. Academic Emergency Medicine. 2016;23:543-553
92. Slessor D, Hunter S. To be blunt: are we wasting our time? Emergency department thoracotomy following blunt trauma: a systematic review and meta-analysis. Annals of Emergency Medicine. 2015;65(3):297-307
93. Slobogean GP, Sprague SA, Scott T, et al. Complications following young femoral neck fractures. Injury. 2015;46:484-491
94. Smith CM, Colquhoun MC. Out-of-hospital cardiac arrest in schools: a systematic review. Resuscitation. 2015;96:296-302
95. Spanos K, Karathanos C, Stamoulis K, et al. Endovascular treatment of traumatic internal carotid artery pseudoaneurysm. Injury. 2016;47:307-312
96. Stang AS, Crotts J, Johnson DW, et al. Crowding measures associated with the quality of emergency department care: a systematic review. Academic Emergency Medicine. 2015;22:643-656
97. Strudwick K, Nelson M, Martin-Khan M, et al. Quality indicators for musculoskeletal injury management in the emergency department: a systematic review. Academic Emergency Medicine. 2015;22:127-141
98. Subramaniam S, Bober J, Chao J, et al. Point-of-care ultrasound for diagnosis of abscess in skin and soft tissue infections. Academic Emergency Medicine. 2016;23:1296-1306
99. Talikowska M, Tohira H, Finn J. Cardiopulmonary resuscitation quality and patient survival outcome in cardiac arrest: a systematic review and meta-analysis. Resuscitation. 2015;96:66-77
100. Unsworth A, Curtis K, Asha SE. Treatment for blunt chest trauma and their impact on patient outcomes and health service delivery. Scandinavian Journal of Trauma, Resuscitation and Emergency Medicine. 2015; 23:17

101. Vargas M, Servillo G, Sutherasan Y, et al. Effects of in-hospital low targeted temperature after out of hospital cardiac arrest: a systematic review with meta-analysis of randomized clinical trials. Resuscitation. 2015;91:8-18
102. Veigas PV, Callum J, Rizoli S, et al. A systematic review on the rotational thrombelastometry (ROTEM) values for the diagnosis of coagulopathy, prediction and guidance of blood transfunsion and prediction of mortality in trauma patients. Scandinavian Journal of Trauma, Resuscitation and Emergency Medicine. 2016;24:114
103. Villa-Roel C, Nikel T, Ospina TNM, et al. Effectiveness of educational interventions to increase primary care follow-up for adults seen in emergency department for acute asthma: a systematic review and meta-analysis. Academic Emergency Medicine. 2016;23:5-13
104. Vrablik ME, Snead GR, Minnigan HJ, et al. The diagnostic accuracy of bedside ocular ultrasonography for the diagnosis of retinal detachment: a systematic review and meta-analysis. Annals of Emergency Medicine. 2015;65(2):199-203
105. Wang RC, Bent S, Weber E, et al. The impact of clinical decision rules on computed tomography used and yield for pulmonary embolism: a systematic review and meta-analysis. Annals of Emergency Medicine. 2016;67(6):693-701
106. West S, Soar J, Callaway CW. The viability of transplanting organs from donor who underwent cardiopulmonary resuscitation: a systematic review. Resuscitation. 2016;108:27-33
107. Williams TA, Tohira H, Finn J, et al. The ability of early warning scores (EWS) to detect critical illness in the prehospital setting: a systematic review. Resuscitation. 2016;102:35-43
108. Yan JW, McLeod SL, Iansavitchene A. Ketamine-propofol versus propofol alone for procedural sedation in the emergency department: a systematic review and meta-analysis. Academic Emergency Medicine. 2015;22:1003-1013
109. Young KD, Korotzer NC. Weight estimation methods in children: a systematic review. Annals of Emergency Medicine. 2016;68(4):441-451
110. Zhang W, Chen E, Xue D, et al. Risk factors for wound complications of closed calcaneal fractures after surgery: a systematic review and meta-analysis. Scandinavian Journal of Trauma, Resuscitation and Emergency Medicine. 2015;23:18
111. Zhang B, Zhu Y, Zhang F, et al. Meta-analysis of plate fixation versus intramedullary fixation for the treatment of mid-shaft clavicle fractures. Scandinavian Journal of Trauma, Resuscitation and Emergency Medicine. 2015;23:27
112. Zhao L, Li C, Liu B, et al. The association of gasping and outcome, in out of hospital cardiac arrest: a systematic review and meta-analysis. Resuscitation. 2015;97:7-12
