# Supplementary material for: Quality of reporting of systematic reviews and meta-analyses in emergency medicine based on the PRISMA statement
Source: BMC Emerg Med. 2019 Feb 11;19:19. doi: 10.1186/s12873-019-0233-6 (PMC6371507; doi:10.1186/s12873-019-0233-6)
Supplement: Supplementary file 4 — PRISMA assessment of each individual review. Final scores for all reviews based on each individual idem of the PRISMA statement. *Full references can be found in Additional file 3. (PDF 976 kb) [file 12873_2019_233_MOESM4_ESM.pdf]

PRISMA assessment of each individual review

Journals 2015	Resuscitation															
**Articles (Reference*)**	Benoit (9)	Bhate (10)	Bougouin (12)	Cheng (21)	Chou (23)	Descatha (29)	Gates (37)	Huang (44)	Mockford (63)	Olaussen (68)	Reynolds (79)	Roberts (80)	Smith (94)	Talikowska (99)	Vargas (101)	Zhao (112)
Article states adherence to PRISMA statement	0	1	0	1	1	1	0	1	0	1	1	0	1	0	1	0
1 Title	1	1	1	1	1	1	1	1	1	1	1	1	1	1	1	1
2 Structured summary	1	1	1	1	1	1	1	1	1	1	1	1	1	1	1	1
3 Rationale	1	1	1	1	1	1	1	1	1	1	1	1	1	1	1	1
4 Objectives	1	1	1	1	1	1	1	1	1	1	1	0	0	1	1	1
5 Protocol and registration	0	0	0	0	0	0	1	0	0	0	0	0	0	1	0	0
6 Eligibility criteria	0	1	1	1	1	1	1	1	1	1	1	1	1	1	1	1
7 Information sources	1	1	1	1	1	1	1	1	1	1	1	0	1	1	1	1
8 Search	1	1	1	1	1	1	0	0	1	0	1	0	0	1	0	0
9 Study selection	0	1	1	1	1	1	1	0	1	1	1	1	1	1	1	1
10 Data collection process	1	0	1	1	1	0	0	1	1	0	1	1	0	1	1	1
11 Data items	1	1	1	1	1	1	1	1	1	1	0	1	1	1	0	1
12 Risk of bias in individual studies	1	1	1	1	1	1	1	1	1	0	1	1	0	1	1	1
13 Summary measures	1	0	1	1	1	1	1	1	0	0	1	0	0	1	1	1
14 Synthesis of results	1	1	1	1	1	1	1	1	1	0	1	1	1	1	1	1
15 Risk of bias across studies	1	0	1	0	1	1	0	0	0	0	0	0	0	0	1	1
16 Additional analyses	1	na	1	0	1	1	1	1	na	na	0	na	na	1	1	1
17 Study selection	0	0	1	0	0	0	0	0	0	0	0	1	0	0	0	1
18 Study characteristics	1	1	1	1	1	1	1	1	1	1	1	1	1	1	1	1
19 Risk of bias within studies	1	0	1	1	1	0	1	1	0	0	1	0	1	1	1	1
20 Results of individual studies	1	1	1	1	1	1	1	1	0	1	1	1	1	1	1	1
21 Synthesis of results	1	1	1	1	1	1	1	1	1	1	1	1	1	1	1	1
22 Risk of bias across studies	na	0	1	0	1	1	0	1	0	0	0	0	0	1	1	1
23 Additional analysis	1	na	1	0	1	0	0	1	na	na	0	na	na	1	1	1
24 Summary of evidence	1	1	1	1	1	1	1	1	1	1	1	1	1	1	1	1
25 Limitations	1	1	1	1	1	1	1	1	1	1	1	1	1	1	1	1
26 Conclusions	1	1	1	1	1	1	0	1	0	1	1	1	0	1	1	1
27 Funding	0	1	0	1	1	1	1	1	1	1	1	1	1	1	1	1
**Percentage**	81	72	93	78	93	81	74	81	68	60	74	68	60	93	85	93
**Total**	21	18	25	21	25	22	20	22	17	15	20	17	15	25	23	25

Journals 2015	Annals of Emergency Medicine					Academic Emergency Medicine								
**Articles (Reference*)**	Asha (2)	Cohen (24)	Juurlink (50)	Slessor (92)	Vrablik (104)	Carpenter (16)	Davis (27)	Galipeau (35)	Oto (71)	Shopp (89)	Sin (90)	Stang (92)	Strudwick (97)	Yan (108)
Article states adherence to PRISMA statement	0	0	0	0	0	1	0	1	0	1	0	1	1	0
1 Title	1	1	1	1	1	1	1	0	0	1	0	1	1	1
2 Structured summary	1	1	1	1	0	1	1	1	1	1	1	1	1	1
3 Rationale	1	1	1	1	1	1	1	1	1	1	1	1	1	1
4 Objectives	1	1	0	1	1	1	1	1	1	1	1	1	1	1
5 Protocol and registration	0	0	0	0	0	0	0	1	0	1	0	0	1	0
6 Eligibility criteria	1	1	0	1	1	1	1	1	1	1	1	1	1	1
7 Information sources	1	1	1	1	1	1	1	1	1	1	1	1	1	1
8 Search	1	1	0	1	1	1	1	1	1	1	0	1	1	1
9 Study selection	1	1	0	1	0	1	1	1	0	1	0	1	1	1
10 Data collection process	1	1	0	1	1	1	1	1	0	0	0	1	1	1
11 Data items	1	1	0	0	0	1	0	1	0	0	0	1	1	1
12 Risk of bias in individual studies	1	1	0	1	1	1	1	1	0	1	1	1	0	1
13 Summary measures	0	0	0	0	1	1	1	1	0	1	0	0	1	1
14 Synthesis of results	1	1	0	1	1	1	1	1	1	1	0	0	1	1
15 Risk of bias across studies	1	0	0	0	0	1	0	0	0	1	0	0	0	0
16 Additional analyses	1	na	na	0	0	1	0	1	na	1	1	na	na	1
17 Study selection	1	0	0	1	0	0	0	0	0	1	0	0	0	1
18 Study characteristics	1	1	1	1	1	1	0	1	1	1	1	1	1	1
19 Risk of bias within studies	1	1	0	1	1	1	1	0	0	0	1	1	0	1
20 Results of individual studies	1	1	1	1	1	1	1	1	1	1	1	1	1	1
21 Synthesis of results	1	1	1	1	1	1	1	1	1	1	1	1	1	1
22 Risk of bias across studies	1	1	0	0	0	na	0	0	0	1	0	0	0	0
23 Additional analysis	na	na	na	0	0	0	1	1	na	1	na	na	na	0
24 Summary of evidence	1	1	1	1	1	1	1	1	1	1	1	1	1	1
25 Limitations	1	1	1	1	1	1	1	1	1	1	1	1	1	1
26 Conclusions	0	1	0	1	1	1	1	1	0	0	1	1	1	1
27 Funding	1	1	1	1	1	1	1	1	1	1	1	1	1	0
**Percentage**	88	84	40	74	67	88	74	81	52	85	58	76	80	81
**Total**	23	21	10	20	18	23	20	22	13	23	15	19	20	22

Journals 2015	Scandinavian Journal of Trauma, Resuscitation and Emergency Medicine						Injury															
**Articles (Reference*)**	Hyldmo (45)	Hyldmo (46)	Leonard (53)	Unsworth (100)	Zhang (110)	Zhang (111)	Ali (1)	Aurégan (3)	Barquet (6)	Doleman (31)	Ghayoumi (38)	Hajibandeh (40)	Harmsen (41)	Marufu (56)	McQueen (59)	O'Dochartaigh (67)	Oteir (70)	Papakostidis (72)	Peters (74)	Ruff (81)	Shen (88)	Slobogean (93)
Article states adherence to PRISMA statement	1	1	1	0	1	1	1	1	1	0	0	1	1	1	1	1	0	1	0	0	1	0
1 Title	1	1	0	0	1	1	1	1	0	1	0	1	1	1	1	1	1	1	1	1	1	0
2 Structured summary	1	1	0	0	1	1	1	1	1	1	0	1	1	1	1	1	1	0	1	1	1	1
3 Rationale	1	1	1	1	1	1	1	1	1	1	1	1	1	1	1	1	1	1	1	1	1	1
4 Objectives	1	1	1	1	1	1	1	1	1	1	1	1	0	1	1	1	1	1	1	1	1	1
5 Protocol and registration	1	1	0	0	0	0	0	0	0	0	0	0	0	0	1	1	1	0	0	0	0	0
6 Eligibility criteria	1	1	1	1	1	1	1	1	1	1	1	1	1	1	1	1	1	0	1	1	1	1
7 Information sources	1	1	0	1	1	1	1	1	1	1	0	1	1	1	1	1	1	1	1	1	1	1
8 Search	1	1	1	1	1	0	0	0	0	0	0	0	1	0	1	0	0	1	0	0	0	1
9 Study selection	1	1	0	0	0	1	0	1	0	0	1	1	1	1	1	1	1	0	1	1	1	1
10 Data collection process	1	1	0	0	1	0	0	1	0	0	0	1	0	1	1	1	1	0	1	0	1	1
11 Data items	1	1	1	0	1	1	0	1	1	1	1	1	1	0	0	1	0	0	1	0	1	1
12 Risk of bias in individual studies	1	1	0	1	1	1	0	1	0	1	0	1	1	1	1	1	1	1	1	1	1	0
13 Summary measures	1	1	0	0	1	1	0	1	0	1	1	1	0	1	0	0	0	1	0	0	1	1
14 Synthesis of results	1	1	0	1	1	1	0	0	1	1	1	1	1	1	0	1	1	1	0	0	1	1
15 Risk of bias across studies	0	0	0	0	1	1	0	1	0	1	0	1	0	0	0	0	0	0	0	0	1	0
16 Additional analyses	na	0	na	na	1	1	na	na	na	1	0	1	na	na	na	na	na	1	na	na	1	1
17 Study selection	0	0	1	0	0	0	1	1	0	0	1	0	0	1	0	0	0	0	0	0	1	0
18 Study characteristics	0	0	1	1	1	1	1	0	1	1	1	1	1	1	1	1	1	1	1	0	1	1
19 Risk of bias within studies	0	0	0	0	1	1	0	0	0	0	0	1	1	0	1	1	0	0	1	0	0	0
20 Results of individual studies	1	1	1	0	1	1	1	0	1	1	1	1	1	1	1	1	1	1	1	0	1	1
21 Synthesis of results	1	1	1	1	1	1	1	1	1	1	1	1	1	1	1	1	1	1	1	1	1	1
22 Risk of bias across studies	0	0	0	0	1	1	0	0	0	1	0	1	0	0	1	1	0	1	0	0	1	0
23 Additional analysis	na	0	na	na	1	1	na	na	na	0	0	1	na	na	na	na	na	0	na	na	1	1
24 Summary of evidence	1	1	1	1	1	1	1	1	1	1	1	1	1	1	1	1	1	1	1	1	1	1
25 Limitations	1	1	1	1	1	1	1	1	1	1	1	1	1	1	1	1	1	1	1	1	1	1
26 Conclusions	0	0	1	1	1	1	1	0	0	1	1	1	1	1	1	1	1	0	1	1	1	1
27 Funding	1	1	0	0	1	1	1	1	0	1	0	1	0	1	1	0	0	0	0	1	1	1
**Percentage**	76	70	48	48	89	85	56	68	48	74	52	89	68	76	80	80	68	56	68	52	89	74
**Total**	19	19	12	12	24	23	14	17	12	20	14	24	17	19	20	20	17	15	17	13	24	20

*Full references can be found in Appendix 3.

PRISMA assessment of each individual review

Journals 2016

Resuscitation

Articles (Reference*)	Buis (13)	Cartledge (17)	Chapman (20)	Chopra (22)	Couper (25)	Hsieh (43)	Inácio (47)	Kim (51)	Millin (62)	Ortega-Deballon (69)	Patel (73)	Quan (76)	Sanfilippo (83)	Schenone (84)	West (106)	Williams (107)
Article states adherence to PRISMA statement	1	1	0	1	0	1	1	0	1	0	1	0	1	1	1	1
1 Title	1	1	0	1	1	1	1	1	1	1	1	1	1	1	1	0
2 Structured summary	0	1	1	1	1	1	1	1	1	1	1	1	1	1	1	1
3 Rationale	1	1	1	1	1	1	1	1	1	1	1	1	1	1	1	1
4 Objectives	1	1	1	1	0	0	1	1	1	1	1	0	0	1	1	1
5 Protocol and registration	0	1	0	0	1	0	0	0	0	0	0	0	0	0	0	1
6 Eligibility criteria	1	1	1	1	1	1	1	1	0	1	1	1	0	1	1	1
7 Information sources	0	1	1	1	0	1	1	1	1	0	1	0	1	1	1	1
8 Search	1	0	1	1	0	1	0	1	0	1	1	1	0	0	1	0
9 Study selection	1	1	1	1	1	1	1	1	1	1	1	1	1	1	1	1
10 Data collection process	0	1	0	0	1	1	1	1	0	1	0	1	0	1	0	0
11 Data items	1	1	1	0	0	1	0	1	0	1	1	1	0	1	1	0
12 Risk of bias in individual studies	1	1	1	1	1	1	1	1	1	1	0	1	1	1	1	1
13 Summary measures	1	0	1	0	1	0	1	1	1	0	0	1	1	0	0	1
14 Synthesis of results	1	0	0	1	1	0	1	1	1	1	0	1	1	1	0	0
15 Risk of bias across studies	0	0	0	0	0	0	1	1	0	0	0	1	0	1	0	1
16 Additional analyses	na	na	0	0	0	na	1	1	0	na	na	0	1	1	na	1
17 Study selection	0	0	0	0	0	0	0	1	0	1	0	0	0	0	0	0
18 Study characteristics	1	1	1	1	1	1	1	1	1	1	1	1	0	1	0	1
19 Risk of bias within studies	1	1	1	0	1	1	0	1	1	0	0	1	1	1	1	1
20 Results of individual studies	1	0	1	0	1	1	1	1	1	1	1	1	1	1	1	1
21 Synthesis of results	1	1	1	1	1	1	1	1	1	1	1	1	1	1	1	1
22 Risk of bias across studies	0	0	0	0	0	0	0	1	0	0	0	1	0	1	0	1
23 Additional analysis	na	na	0	0	0	na	1	1	0	na	na	0	1	1	na	0
24 Summary of evidence	1	1	1	1	1	1	1	1	1	1	1	1	1	1	1	1
25 Limitations	1	0	1	1	1	0	1	1	0	1	0	1	0	1	1	0
26 Conclusions	1	1	1	1	1	1	1	1	1	1	1	1	0	1	0	1
27 Funding	0	1	1	1	1	1	1	1	1	1	1	1	1	1	1	1
**Percentage**	68	68	67	59	67	68	78	96	59	76	60	78	56	85	64	70
**Total**	17	17	18	16	18	17	21	26	16	19	15	21	15	23	16	19

Annals of Emergency Medicine / Academic Emergency Medicine

Articles (Reference*)	Annals of Emergency Medicine: Bonnes (11)	Chaikriangkrai (18)	Raven (78)	Wang (105)	Young (109)	Academic Emergency Medicine: Bellolio (8)	Carpenter (15)	Chao (19)	Cournoyer (26)	Eliyahu (34)	Hartling (42)	Martindale (55)	Menon (60)	Ramaekers (77)	Sin (90)	Subramaniam (98)	Villa-Roel (103)
Article states adherence to PRISMA statement	1	0	1	1	0	1	1	0	1	1	0	1	0	0	1	1	1
1 Title	1	1	1	1	1	1	1	1	1	1	0	1	1	0	0	0	1
2 Structured summary	1	1	1	1	1	1	1	1	1	1	1	1	1	1	1	1	1
3 Rationale	1	1	1	1	1	1	1	1	1	1	1	1	1	1	1	1	1
4 Objectives	0	1	1	1	0	1	1	1	1	1	1	1	1	1	1	1	1
5 Protocol and registration	0	0	1	1	0	0	0	0	0	1	0	0	0	0	0	0	0
6 Eligibility criteria	1	1	1	1	1	1	1	1	1	1	0	1	1	1	1	1	0
7 Information sources	1	1	0	0	0	1	1	0	1	1	1	1	1	0	1	1	1
8 Search	0	0	1	1	0	1	1	0	1	1	0	0	0	1	0	1	1
9 Study selection	0	1	1	1	1	1	1	1	1	1	1	1	1	1	1	1	1
10 Data collection process	1	1	0	1	0	1	0	1	0	1	1	1	1	1	1	1	0
11 Data items	0	1	1	1	1	1	1	1	0	1	0	1	0	1	0	1	0
12 Risk of bias in individual studies	1	1	1	1	0	1	1	1	1	1	1	1	0	1	1	1	1
13 Summary measures	1	1	0	1	1	1	1	0	1	1	1	1	0	1	0	1	1
14 Synthesis of results	1	1	1	1	1	1	1	1	1	1	1	1	0	1	0	1	1
15 Risk of bias across studies	1	1	0	1	0	1	1	0	1	0	0	1	0	0	0	0	0
16 Additional analyses	1	1	na	1	na	0	1	1	1	na	na	0	na	1	na	0	1
17 Study selection	0	0	1	0	1	1	0	0	0	0	0	0	1	0	0	0	0
18 Study characteristics	1	1	1	1	1	1	1	1	1	1	1	1	1	1	1	1	1
19 Risk of bias within studies	1	0	0	1	0	1	1	1	1	1	1	0	0	1	1	1	1
20 Results of individual studies	1	1	1	1	1	1	1	1	1	1	1	1	1	1	1	1	1
21 Synthesis of results	1	1	1	1	1	1	1	1	1	1	1	1	1	1	1	1	1
22 Risk of bias across studies	1	1	0	1	0	0	na	0	1	0	0	na	0	0	0	0	1
23 Additional analysis	1	1	na	1	na	1	1	1	1	na	na	0	na	1	na	0	0
24 Summary of evidence	1	1	1	1	1	1	1	1	1	1	1	1	1	1	1	1	1
25 Limitations	1	1	1	0	1	1	1	1	0	1	1	1	1	1	1	1	1
26 Conclusions	1	0	1	1	0	0	1	1	1	1	0	1	1	1	1	0	1
27 Funding	1	1	1	1	1	1	1	1	1	1	1	1	1	1	1	1	1
**Percentage**	78	81	76	89	60	85	88	74	81	88	64	77	64	78	64	70	74
**Total**	21	22	19	24	15	23	23	20	22	22	16	20	16	21	16	19	20

Scandinavian Journal of Trauma, Resuscitation and Emergency Medicine

Articles (Reference*)	Bakhshayesh (5)	Deslarzes (30)	Ekmejian (34)	Jiang (49)	Li (54)	Mikalsen (61)	Veigas (102)
Article states adherence to PRISMA statement	1	0	1	1	0	0	1
1 Title	1	0	1	1	1	0	1
2 Structured summary	1	1	1	1	1	0	1
3 Rationale	1	1	1	1	1	1	1
4 Objectives	1	1	1	1	1	1	0
5 Protocol and registration	0	0	0	1	0	0	0
6 Eligibility criteria	1	1	1	1	1	1	1
7 Information sources	0	1	1	1	1	1	1
8 Search	0	1	1	1	0	0	1
9 Study selection	1	0	1	1	1	0	1
10 Data collection process	0	0	0	0	1	0	1
11 Data items	0	1	0	1	0	0	1
12 Risk of bias in individual studies	0	0	0	1	0	0	1
13 Summary measures	0	1	0	1	1	0	0
14 Synthesis of results	0	1	0	1	1	0	0
15 Risk of bias across studies	0	0	0	1	1	0	0
16 Additional analyses	na	1	na	1	0	na	na
17 Study selection	0	1	0	0	1	1	0
18 Study characteristics	0	0	1	1	0	1	1
19 Risk of bias within studies	0	0	1	1	1	na	1
20 Results of individual studies	0	0	1	1	1	1	1
21 Synthesis of results	1	1	1	1	1	1	1
22 Risk of bias across studies	0	0	0	1	1	0	0
23 Additional analysis	na	1	na	1	1	na	na
24 Summary of evidence	0	1	1	1	1	0	1
25 Limitations	0	1	1	1	1	0	1
26 Conclusions	1	1	1	1	0	1	1
27 Funding	1	0	1	1	1	0	1
**Percentage**	36	59	64	93	74	38	72
**Total**	9	16	16	25	20	9	18

Injury

Articles (Reference*)	Aurégan (4)	Bartlett (7)	Camarda (14)	Dekker (28)	Edwards (32)	Garara (36)	Guest (39)	Inge (48)	Laan (52)	McCarthy (57)	McMahon (58)	Morelli (64)	Nunn (65)	Nyholm (66)	Peeters (74)	Sammy (82)	Schimmer (85)	Sharples (86)	Sharrock (87)	Spanos (95)
Article states adherence to PRISMA statement	1	0	1	1	1	1	0	0	1	0	1	1	1	0	0	1	0	1	1	0
1 Title	1	1	1	1	1	1	1	1	1	0	1	1	1	1	1	1	1	0	1	0
2 Structured summary	0	1	1	1	1	1	1	1	1	1	1	1	1	1	1	1	1	1	1	1
3 Rationale	1	1	1	1	1	1	1	1	1	1	1	1	1	1	1	1	1	1	1	1
4 Objectives	1	1	1	1	1	1	1	1	1	0	1	1	1	1	1	1	1	1	1	1
5 Protocol and registration	1	0	0	0	0	1	0	0	0	0	0	0	1	1	0	0	0	0	0	0
6 Eligibility criteria	1	1	1	1	1	1	1	1	1	1	1	1	1	1	1	1	1	1	1	0
7 Information sources	1	1	0	1	0	1	1	1	1	0	1	1	1	1	1	1	1	1	1	1
8 Search	0	0	0	1	0	1	0	0	1	1	0	0	1	0	0	1	0	0	1	0
9 Study selection	1	0	1	1	0	1	1	1	1	0	1	1	1	1	1	0	1	0	1	0
10 Data collection process	1	1	1	1	0	0	0	0	0	0	1	0	1	1	1	0	0	0	1	0
11 Data items	1	1	1	1	1	1	0	0	0	0	1	1	1	1	1	0	1	1	1	1
12 Risk of bias in individual studies	1	0	1	1	0	1	1	0	0	1	1	0	1	0	1	1	1	1	1	0
13 Summary measures	1	0	0	1	0	0	0	0	1	1	0	0	1	0	1	1	0	1	0	0
14 Synthesis of results	1	0	1	1	1	0	0	0	0	0	1	0	1	0	0	0	0	1	1	1
15 Risk of bias across studies	0	0	0	0	0	0	0	0	0	0	0	0	0	0	0	0	0	0	0	0
16 Additional analyses	1	na	na	0	0	na	na	na	0	na	na	0	na	na	na	1	na	0	0	na
17 Study selection	1	1	0	0	0	0	1	1	0	0	0	1	0	1	1	0	0	0	0	0
18 Study characteristics	0	0	1	1	1	1	1	1	1	1	1	1	1	1	1	1	1	1	1	0
19 Risk of bias within studies	0	0	0	0	0	1	1	0	0	0	1	0	1	0	1	0	1	1	1	0
20 Results of individual studies	0	1	1	1	1	0	0	1	1	0	1	0	1	0	0	1	1	1	1	0
21 Synthesis of results	1	1	1	1	1	1	1	1	1	1	1	0	1	0	1	1	1	1	1	1
22 Risk of bias across studies	0	0	0	0	0	0	0	0	0	0	0	0	0	0	0	0	0	0	0	0
23 Additional analysis	na	na	na	1	0	na	na	na	0	na	na	0	na	na	na	1	na	0	0	na
24 Summary of evidence	1	1	1	1	1	1	1	1	1	1	1	1	1	1	1	1	1	1	1	1
25 Limitations	1	1	1	0	1	1	1	0	1	0	1	1	0	1	0	1	0	1	1	1
26 Conclusions	0	0	1	0	0	1	1	1	0	1	1	1	0	0	1	1	1	1	0	1
27 Funding	1	0	0	1	0	1	1	1	1	0	1	1	1	1	0	0	0	0	0	0
**Percentage**	69	52	64	70	44	72	64	56	56	40	76	52	80	60	68	63	60	59	67	40
**Total**	18	13	16	19	12	18	16	14	15	10	19	14	20	15	17	17	15	16	18	10

*Full references can be found in Appendix 3.
